# Genetic predisposition to neural crest-derived tumors: revisiting the role of *KIF1B*

**DOI:** 10.1530/EC-20-0460

**Published:** 2020-10-08

**Authors:** Catherine Cardot Bauters, Emmanuelle Leteurtre, Bruno Carnaille, Christine Do Cao, Stéphanie Espiard, Malo Penven, Evelyne Destailleur, Isabelle Szuster, Tonio Lovecchio, Julie Leclerc, Fredéric Frénois, Emmanuel Esquivel, Patricia L M Dahia, Emilie Ait-Yahya, Michel Crépin, Pascal Pigny

**Affiliations:** 1 CHU Lille, Service d’Endocrinologie, Diabétologie, Métabolisme-Nutrition, Hôpital Claude Huriez, Lille, France; 2 Univ. Lille, Inserm, CHU Lille, UMR-S 1277-CANTHER, Cancer Heterogeneity, Plasticity & Resistance to Therapies, Lille, France; 3 CHU Lille, Service de Chirurgie Endocrine, Hôpital Claude Huriez, Lille, France; 4 CHU Lille, Service de Biochimie Hormonologie, Métabolisme, Nutrition-Oncologie, Centre de Biologie Pathologie Génétique, Lille, France; 5 Univ. Lille, CHU Lille, EA-7364 RADEME, Faculté de Médecine, Lille, France; 6 Dept Medicine, Mays Cancer Center, University of Texas Health Science Center at San Antonio, San Antonio, Texas, USA; 7 CHU Lille, Institut de Biochimie & Biologie Moléculaire, Centre de Biologie Pathologie Génétique, Lille, France

**Keywords:** endocrine tumor, genetic predisposition, pheochromocytoma, neural crest

## Abstract

**Objective:**

We previously described a family in which predisposition to pheochromocytoma (PCC) segregates with a germline heterozygous *KIF1B* nucleotide variant (c.4442G>A, p.Ser1481Asn) in three generations. During the clinical follow-up, one proband’s brother, negative for the *KIF1B* nucleotide variant, developed a bilateral PCC at 31 years. This prompted us to reconsider the genetic analysis.

**Design and methods:**

Germline DNA was analyzed by next-generation sequencing (NGS) using a multi-gene panel plus MLPA or by whole exome sequencing (WES). Tumor-derived DNA was analyzed by SnapShot, Sanger sequencing or NGS to identify loss-of-heterozygosity (LOH) or additional somatic mutations.

**Results:**

A germline heterozygous variant of unknown significance in *MAX* (c.145T>C, p.Ser49Pro) was identified in the proband’s brother. Loss of the wild-type *MAX* allele occurred in his PCCs thus demonstrating that this variant was responsible for the bilateral PCC in this patient. The proband and her affected grandfather also carried the *MAX* variant but no second hit could be found at the somatic level. No other pathogenic mutations were detected in 36 genes predisposing to familial PCC/PGL or familial cancers by WES of the proband germline. Germline variants detected in other genes, *TFAP2E* and *TMEM214*, may contribute to the multiple tumors of the proband.

**Conclusion:**

In this family, the heritability of PCC is linked to the MAX germline variant and not to the *KIF1B* germline variant which, however, may have contributed to the occurrence of neuroblastoma (NB) in the proband.

## Introduction

We previously described a family in which predisposition to pheochromocytoma (PCC), segregates into three generations with a germline heterozygous nucleotide variant of *KIF1B* (c.4442G>A, p.Ser1481Asn) which encodes the kinesin-like protein KIF1B (1). KIF1B isoform β (KIF1Bβ) is a molecular motor protein that participates in the transport of synaptic vesicle precursors and is essential for neuronal survival and differentiation (2). *In vitro*, the p.Ser1481Asn variant decreases the ability of KIF1Bβ to promote the apoptosis of primary rat sympathetic neurons (3) and thus may facilitate tumorigenesis later on. Conversely enforced expression of KIF1B resulted in an induction of apoptosis of neuroblastoma (NB) cells (4). Thus, the KIF1Bβ neuronal pro-apoptotic effect combined with the mapping of *KIF1B* on chromosome 1p36, a region frequently deleted in PCC and NB (5), suggested that *KIF1B* might function as a tumor suppressor gene (TSG) in these diseases (3). However, in our kindred, we did not identify a loss of the wild type (WT) allele of* KIF1B* at the somatic level in the PCC or NB of the proband, deviating from the Knudson two hits theory (1). At that time, we thus hypothesized that the p.S1481N variant of KIF1Bβ functions in haploinsufficiency in these tumors (1) but its exact mechanisms of action remain unclear (6).

Since our initial report, the large size KIF1B gene has been rarely incorporated in the PCC/PGL gene panels which are analyzed in patients with PCC or PGL by next-generation sequencing to identify familial tumors. Welander *et al.* (7) described one PCC patient with a germline variant of *KIF1B* classified as disease-causing in their cohort thus representing a prevalence of 1.1%. The PCC had a sporadic presentation in this woman who later presented with endometrial carcinoma. Curras-Freixes et al. (8) reported three germline variants of unknown significance (VUS) of *KIF1B* in their cohort of PCC patients, representing a prevalence of 0.66% (3/453) in line with our own estimation of the prevalence of *KIF1B* VUS at 1.3% (1/74 patients with PCC/PGL analyzed by NGS between 2017 and 2019, unpublished data).

In conclusion, only 1–2% of the patients with PCC or PGL had a germline pathogenic variant of *KIF1B* thus leading to frequent questioning on its involvement in the heritability of PCC/PGL (9, 10).

At the time of our report in 2008, the proband’s two brothers, who did not carry the* KIF1B* germline variant, were clinically asymptomatic (1). However, during the clinical follow-up, one of proband’s brother developed a bilateral PCC at 31 years. This prompts us to extend the genetic analysis in this family and to reconsider the molecular pathogenesis of the PCC.

## Materials and methods

### Clinical data

**Figure 1:**
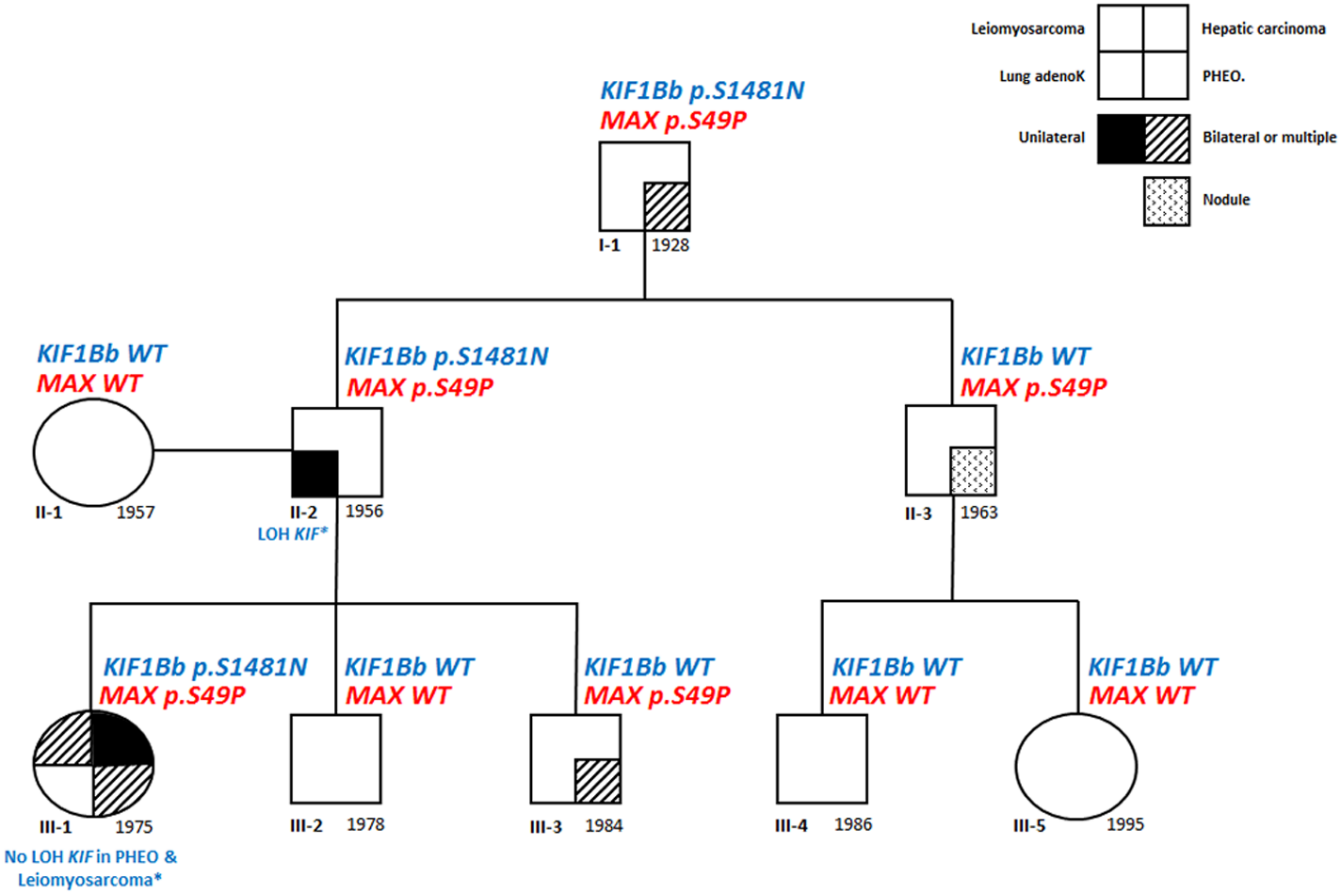
Phenotype and genotype of the family. The proband is patient III.1.

The pedigree of the family has been updated in Fig. 1. In brief, the proband III-1 developed NB of the broad ligament at 17 months which was treated by surgery, radiation therapy and chemotherapy. At age 22 years, she developed a right PCC plus a ganglioneuroma at the site of the original NB and also an ileal schwannoma. At that time, she had hypertension and high normetanephrines levels (exact data not available). Six years later, the patient underwent adrenal surgery for a left PCC associated with a mature ganglioneuroma. At the same time, a well-differentiated leiomyosarcoma arising from the mesosigmoid was detected and surgically removed. At 39 years, several cutaneous metastases of the leiomyosarcoma were surgically removed. At 42 years, a 9-cm moderately differentiated hepatic carcinoma was diagnosed and surgically removed. Finally, at 43 years a uterine leiomyoma, and two metastases (parietal and peritoneal) from the leiomyosarcoma were removed. Her paternal grandfather (I-1) had bilateral PCC at 70 years and her father (II-2) had a lung adenocarcinoma at 47 years and prostatic cancer at 54 years. The proband’s youngest brother (III-3) presented at 31 years with a cardiomyopathy complicated by a Takotsubo’s syndrome which led to the diagnosis of bilateral PCC. The proband’s paternal uncle (II-3) was diagnosed at 56 years with an oligo-symptomatic adrenal nodule exhibiting a high [^18^F]-DOPA uptake at PET.

### Custom endocrine tumors NGS panel

In compliance with the French regulation, each patient gave his/her written informed consent before performing genetic testing which is an integral part of the patient’s care. Moreover, this protocol was reviewed and validated by the Ethical Committee (Comité de Protection des Personnes Nord Ouest IV) under the number HP 20/04. A custom panel based on the hybridization and capture technology (Haloplex, Agilent Technologies) was designed to be compatible with the Illumina MiSeq platform. Probes covered the coding regions and intronic flanking sites of 21 susceptibility genes for endocrine tumors, either PCC/PGL (*VHL*, *RET*, *SDHA*, *SDHAF2*, *SDHB*, *SDHC*, *SDHD*, *TMEM127*, *KIF1B*, *MAX*, *PHD2*) or pituitary tumors/hyperparathyroidism (*MEN1*, *HRPT2*, *AIP*, *CASR*, *CYP24A1*, *GCM2*, *PTH*, *GNA11*, *AP2S1*, *CDKN1B*). Genomic DNA extracted from blood cells was fragmented with restriction enzymes; then digested DNA was hybridized to Haloplex probes which resulted in circularization of DNA fragments and sample indexing. Target DNA was captured with streptavidin-coated magnetic beads, ligated and eluted before bridge PCR amplification of the libraries. After quantification of enriched target DNA, samples were pooled for multiplexed sequencing. NGS sequencing data were aligned to hg19 human reference and annotated using two independent bioinformatic pipelines (alignment using bwa v0.7.15-r1140 followed by best practices for germline variant detection using GATK v3.7 (11) and SeqNext V4.4 (JSI) (12)). Data were filtered using an in-house database (DVD) to remove recurrent sequencing errors. Regions with coverage <30X were reanalyzed using Sanger sequencing.

### Somatic cancer panel analysis

DNA extracted from three separate regions of the frozen left PCC from proband (III-1) was analyzed for somatic nucleotide variants in hot spot regions of 48 commonly mutated cancer genes using the Illumina TruSeq Amplicon Cancer Panel (*ABL1*, *AKT1*, *ALK*, *APC*, *ATM*, *BRAF*, *CDH1*, *CDKN2A*, *CSF1R*, *CTNNB1*, *EGFR*, *ERBB2*, *ERBB4*, *FBXW7*, *FGFR1*, *FGFR2*, *FGFR3*, *FLT3*, *GNA11*, *GNAQ*, *GNAS*, *HNF1A*, *HRAS*, *IDH1*, *JAK2*, *JAK3*, *KDR*, *KIT*, *KRAS*, *MET*, *MLH1*, *MPL*, *NOTCH1*, *NPM1*, *NRAS*, *PDGFRA*, *PIK3CA*, *PTEN*, *PTPN11*, *RB1*, *RET*, *SMAD4*, *SMARCB1*, *SMO*, *SRC*, *STK11*, *TP53*, *VHL*), run on Illumina MiSeq following standard protocols, and analyzed using the filter settings described previously.

### WES (whole exome sequencing) analysis

Genomic DNA extracted from fresh blood cells from the proband and her father with QiAmp DNA mini kit (Qiagen) was captured using Agilent in-solution enrichment methodology with their biotinylated oligonucleotides probes library (SureSelect Clinical Research Exome V2, Agilent Technologies), followed by paired-end 75 bases massively parallel sequencing on Illumina HiSeq4000 (IntegraGen SA, Evry, France), as reported (13). Sequence capture, enrichment and elution were performed according to manufacturer’s instruction and protocols (SureSelect, Agilent) without modification, except for library preparation performed with NEBNext® Ultra kit (New England Biolabs). For library preparation, 600 ng of each genomic DNA were fragmented by sonication and purified to yield fragments of 150–200 bp. Paired-end adaptor oligonucleotides were ligated on repaired fragments then purified and enriched by 8 PCR cycles. A total of 1200ng of the purified Libraries were then hybridized to the SureSelect oligo probe capture library for 72 h. After hybridization and washing, the eluted fraction was PCR-amplified, purified and quantified by qPCR. Each eluted-enriched DNA library was then sequenced on an Illumina HiSeq4000 as paired-end 75 bp reads. Image analysis and base calling were performed using Illumina Real Time Analysis (2.7.7) with default parameters.

### Bioinformatic analysis

After demultiplexing and FASTQ generation, the paired-end reads were trimmed using TrimGalore v0.4.4. The paired-end reads were then aligned to hg19 human reference genome with BWA v0.7.15-r1140 (11). We applied the GATK v3.7 pipeline (12) for indel realignment, duplicate removal, and performed SNP and INDEL discovery as well as share genotype across both the proband and her father’s samples simultaneously according to GATK Best Practices recommendations (14, 15). Variants were annotated and filtered with Agilent Technologies Bench Lab NGS v5.0.2. To select putative pathogenic variants, filters were applied as follows (Supplementary Fig. 1, see section on supplementary materials given at the end of this article): (i) variants common to proband and her father were filtered out; (ii) low quality reads were filtered out; (iii) only heterozygous variants were retained; (iv) variants predicted as pathogenic (Supplementary Fig. 1) were retained; (v) variants in exonic regions or near splicing sites were retained; (vi) variants with a frequency ≥0.01 in the general population (using Exac database, release 0.3, 1000 Genomes Phase1 release v3.20101123, ESP6500SI-V2, 1000 Genomes Phase 3 release v5.20130502) were filtered out. Relevant nucleotide variants were validated by Sanger sequencing on Applied Biosystems 3730 platform.

### Search for large rearrangements

The analysis of large rearrangements was performed with the Multiplex Ligation-dependent Probe Amplification (MLPA) technology for *FH* and *SDH*x genes and by multiplex PCR (QMPSF) for *VHL*. MLPA probes (ref. SALSA MLPA Probemix P198, P226 and P429) and reagents were manufactured and supplied by MRC-Holland (Amsterdam, The Netherlands). The MLPA procedure was conducted according to manufacturer’s specifications. The amplified probes were analyzed on a 3130XL DNA Analyzer (Life Tech, ThermoFisher). Regarding *FH* analysis, the proband DNA sample was tested twice in the same run along with 10 negative samples and one positive control with total deletion of the *FH* gene. Data were interpreted with Coffalyser software (MRC Holland).

### LOH analysis

DNA from formalin-fixed paraffin-embedded (FFPE) tumor samples was extracted using the QIAAmp DNA FFPE kit (Qiagen). Tissue samples of the proband available for LOH analysis are detailed in Supplementary Table 1. LOH of selected nucleotide variant was searched by SNaPshot analysis as previously described (16). PCR and extension primers details are available upon request. Extension products were analyzed on Applied Biosystems 3730 along with GeneScan 120LIZ molecular marker using the Genemapper software. In addition, visualization of the variant peaks on Sanger sequence traces was done using the Mutation Quantifier tool (Surveyor program, Softgenetics).

### Immunohistochemical analysis

Fumarate Hydratase expression was assessed on paraffin-embedded tumors using an anti-FH antibody 1:1000 dilution as previously described (17).

### In silico analysis

Prediction of the missense variant of MAX protein was carried out with the Phyre2 server (Protein Homology/analogY Recognition Engine V2.0). We compared the deduced human amino‐acid 3D structure with the 3D resolved structure of the *Homo sapiens* MAX protein (99% sequence identity) using The PyMOL Molecular Graphics System (v2.0, Schrödinger, LLC). The SuperPose server v.1.0 (18) was used to estimate the structural homology, measuring the average distance between the backbones of superimposed proteins.

## Results

**Figure 2:**
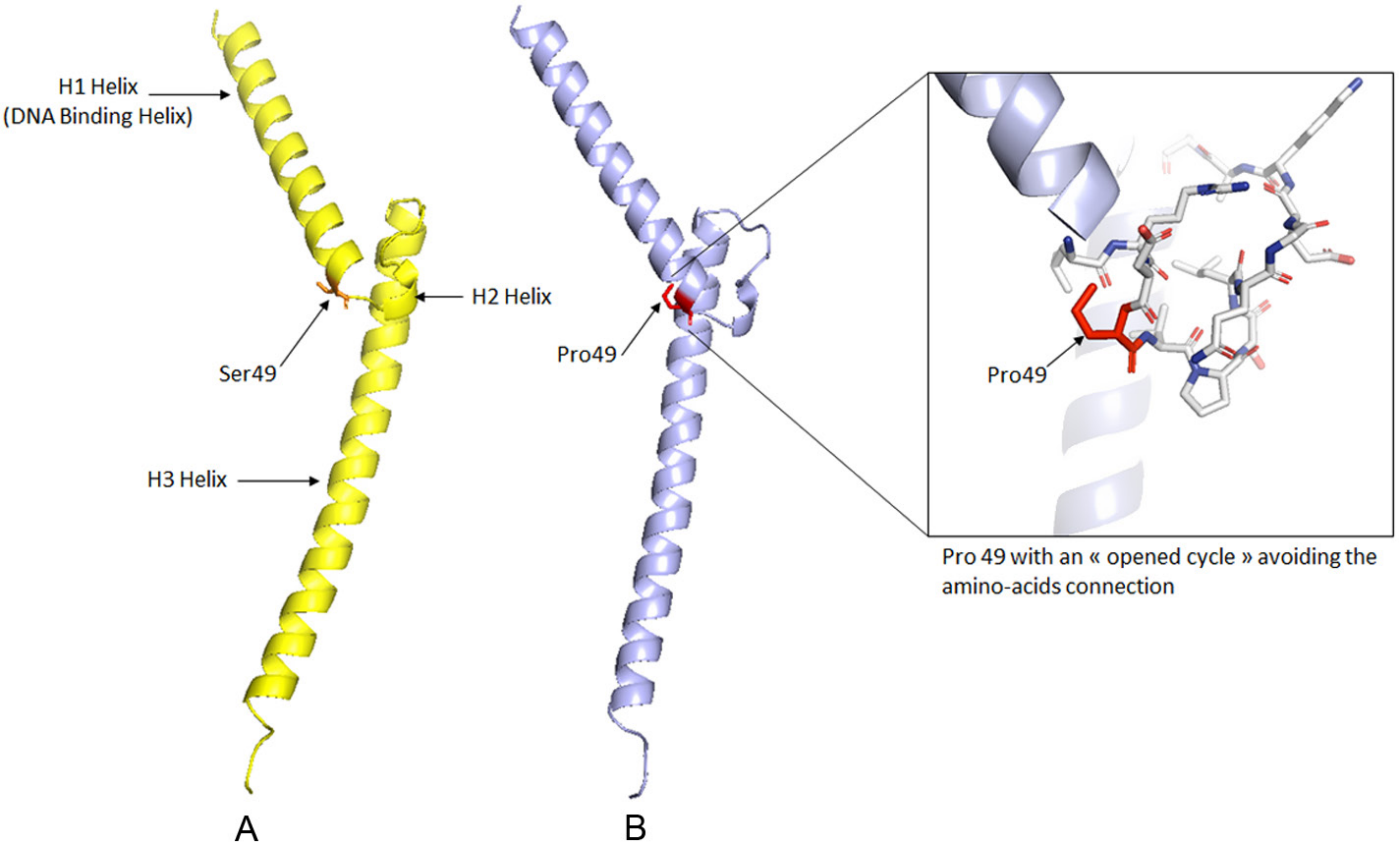
(A) 3D structure of the human native MAX protein (pdb code: 1AN2) (amino acids 22–104); (B) 3D structural prediction of the human Ser49Pro mutated MAX protein (amino acids 22–104).

During the clinical follow-up of the family, patient III-3, who did not harbor the *KIF1B* c.4442G>A nucleotide variant in his germline DNA, developed bilateral PCC at 31 years. His germline DNA was analyzed by NGS using a custom endocrine tumor panel which includes 11 major susceptibility genes for familial PCC/PGL (*PHD2*, *KIF1Bβ*, *SDHA*, *SDHB*, *SDHC*, *SDHD*, *SDHAF2*, *VHL*, *MAX*, *TMEM127* and *RET*). The only heterozygous variant identified was in *MAX (NM_002382.5)*: c.145T>C, p.Ser49Pro. A large rearrangement of *SDHA*, *SDHB*, *SDHC*, *SDHD*, *SDHAF2*, *FH* and *VHL* was excluded by MLPA or QMPSF analysis, respectively. Using the ACMG guidelines and Varsome tool (19), the *MAX* nucleotide variant was classified as a VUS. The secretory profile of his tumors consisted of increased urinary normetanephrines at 1979 nmol/mmol of creatinine (8x the upper limit range) with normal total metanephrines at 182 nmol/mmol of creatinine (<190), reminiscent of the pattern observed in patients with a *MAX* pathogenic variant (20). The p.Ser49Pro MAX variant is predicted to show the Proline 49 with an opened cycle which is impossible, leading probably to the precipitation of the protein (Fig. 2). Proline is a constrained amino acid due to its pyroxylin cycle hindering the rotation of the ϕ angle with the previous amino acid and thus altering the amino acid connection. Therefore, this variant is likely deleterious.

We thus reanalyzed the germline DNAs of the proband (III-1), her father (II-1) and grandfather (I-1), who had been previously found to be carriers of the *KIF1B* c.4442G>A nucleotide variant (1), using the NGS panel. All also carried the *MAX* c.145T>C variant at the heterozygous level. No other pathogenic variant in the nine other susceptibility genes for PCC/PGL was identified (Fig. 1). We also excluded a large rearrangement of *SDHA*, *SDHB*, *SDHC*, *SDHD*, *SDHAF2*, *FH* and *VHL* in the proband by MLPA or QMPSF analysis, respectively. The *MAX* c.145T>C variant was also detected by Sanger sequencing at the heterozygous level in the germline DNA of the proband’s paternal uncle (II-3), who was *KIF1B* negative (Fig. 1). Interestingly, II-3 recently developed, at age 56, a 11-mm hypervascular nodule in his right adrenal which remains clinically silent but strongly uptakes [^18^F]-DOPA during PET, suggestive of PCC or adrenomedullary nodule. Plasma free normetanephrines were at the upper limit of the normal values. Pre-operatively, patient I-1 had high urinary normetanephrines (5x the upper limit range) and chromogranin-A levels (x3 the upper limit range). By contrast, patient II-2, who had a regular follow-up by PET imaging due to his lung and prostatic cancers, never demonstrated an adrenal uptake of [^18^F]-deoxy-glucose. Moreover, his levels of metanepherines/normetanephrines were in the normal range at each follow-up, both elements being in disfavor of a PCC.

**Figure 3:**
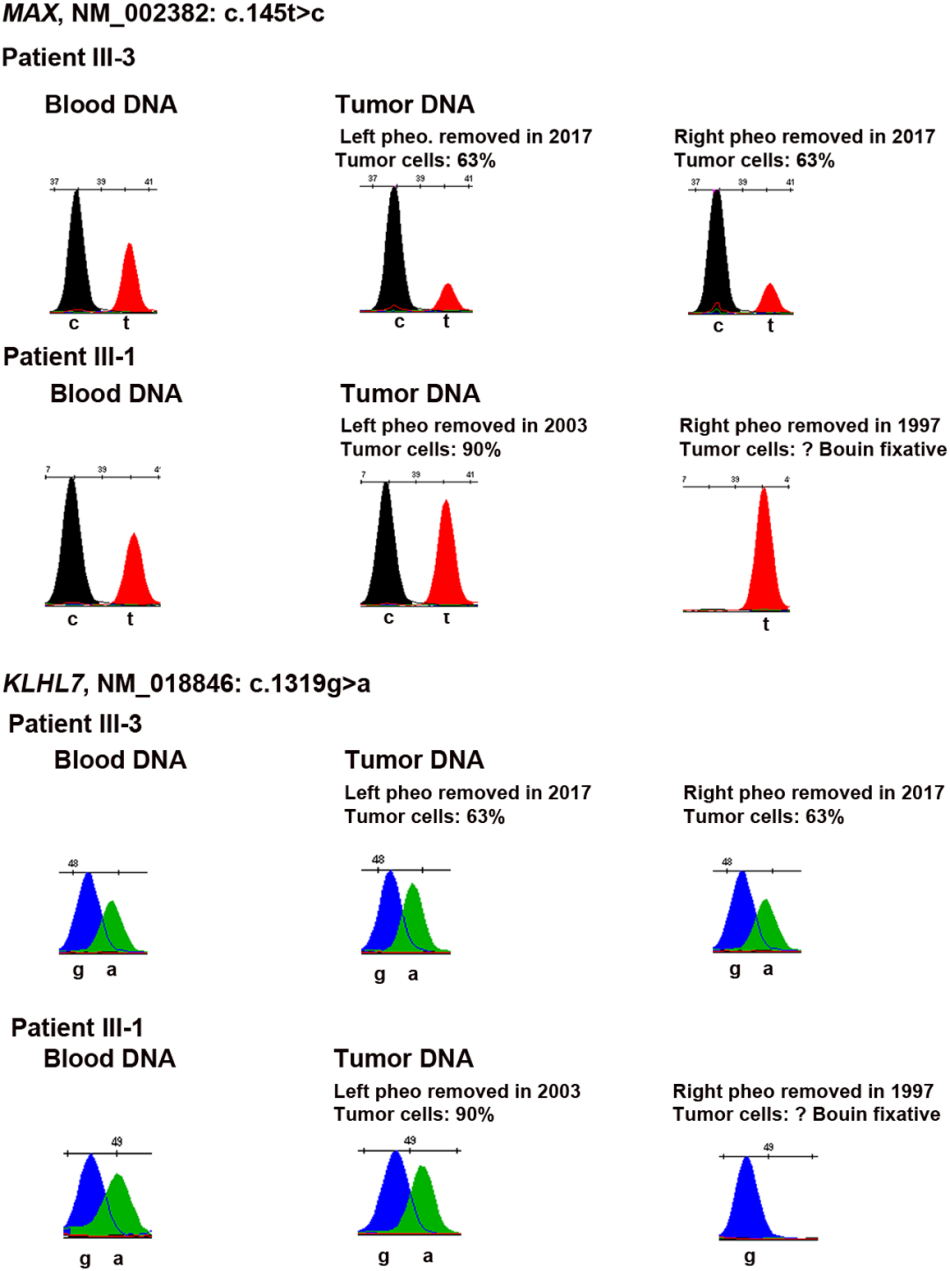
Search for a LOH of *MAX* and *KLHL7* in the DNA extracted from the pheochromocytomas of patients III.1 and III.3 by SnapShot analysis.

Since *MAX* behaves as a TSG (20), we analyzed the tumor DNAs of patient III-3 and identified loss of the *MAX* WT allele in both tumors (Fig. 3) suggesting that this variant is indeed responsible for the bilateral PCC in this patient. In contrast, no LOH of the WT allele of *MAX* was found in the 2 PCCs of the proband (III-1), using DNA obtained from three independent samples, FFPE samples from left and right PCCs (Fig. 3), and one fresh frozen sample from the left PCC (Supplementary Fig. 2). The oldest of the samples (the right PCC) in fact showed complete absence of the variant allele (Fig. 3), which could be due to allelic dropout in the PCR, a well-recognized cause of errors in DNA from suboptimal samples (21). Although the estimated proportion of tumor cells of the left PCC FFPE sample was high (Fig. 3), this estimate was not available from the bulk frozen specimen from this tumor nor the right PCC, so it is unclear to what extent normal cell admixture may have contributed to the allelic count. We also excluded, by Sanger sequencing, the presence of an additional somatic *MAX* mutation, which might have functioned as the second hit in the absence of LOH in the DNA extracted from the left PCC of the proband. We further examined the DNA from the three separate fragments from the left PCC to search for additional somatic mutations in 48 cancer genes using NGS. The three fragments displayed similar variant allele frequencies across these genes, suggesting that the left PCC was homogeneous with respect to both genetic and cell composition. Moreover, these fragments lacked areas of allelic imbalance, in favor of a high level of normal cells in these fragments. Only a few VUS were detected (Supplementary Table 2); however, no variant frequency suggestive of LOH was observed in these three samples. Finally, FH expression evaluated by immunohistochemistry was conserved on the uterine leiomyoma and pelvic leiomyosarcoma of the proband (Supplementary Fig. 3), which strongly suggests the absence of somatic pathogenic variants of *FH* responsible for these 2 tumors in the proband.

Since the proband (III-1) and her father (II-2) shared the same genotype for *MAX* and *KIF1B* despite very different phenotypes (Fig. 1), we considered that the proband may carry additional variants in known/new susceptibility genes that modified cancer predisposition, which might have been inherited from her mother or have occurred *de novo*. WES was performed on the germline DNA of III-1 and II-2. First, the proband had no pathogenic variant in *NF1*, *IDH1*, *IDH2*, *FH*, *MDH2* or *SLC25A11* which predispose to familial PCC/PGL nor in a group of 30 cancer-predisposing genes (22), that is, *APC*, *ATM*, *BAP1*, *BARD1*, *BMPR1A*, *BRCA1*, *BRCA2*, *BRIP1*, *CDH1*, *CDKN2A*, *CDK4*, *CHEK2*, *EPCAM*, *GREM1*, *MITF*, *MLH1*, *MSH2*, *MSH6*, *MUTYH*, *NBN*, *PALB2*, *PMS2*, *POLD1*, *POLE*, *PTEN*, *RAD51C*, *RAD51B*, *SMAD4*, *STK11* and *TP53*. Thus, we decided to focus on the nucleotide variants which were present in the germline of III-1 but absent in II-2 in agreement with our working hypothesis. One hundred twenty-five nucleotide variants were identified as unique to III-1 (Supplementary Fig. 1). Class 1 and 2 variants (benign or probaby benign) were filtered out using Varsome, leading to a list of 24 variants all classified as VUS (Class 3) (Table 1). After the interrogation of several resources such as PUBMED (for a link between the gene of interest and cancer), UniProt (for information on encoded protein function), HGMD (for information on germline mutations currently identified), TCGA (for somatic mutations catalogue), Protein Atlas and CTEX databases (for detailed information on tissue expression), the list was narrowed down to five variants occurring in five genes (Table 1). *KLH7* and *PKM* were good candidate genes for hereditary PCC since they encode proteins expressed in adrenals (23) and are mutated (though rarely) at the somatic level in PCC (TCGA PCPG). *RIPK3*, *TFAP2E* and *TMEM214* were good candidates for the non-neural crest tumors since they are mutated in sarcomas (TCGA SARC), myomatous neoplasms and in hepatocarcinomas (TCGA LIHC) at the somatic level. No protein expression data were available for TMEM214, TFAP2E and RIPK3 in the Protein Atlas (23). Sanger sequencing confirmed the presence of the five nucleotide variants in the germline of the proband and her mother but absence in her father as expected (Table 2), none were *de novo*. Patient III-3 was heterozygous only for the *KLHL7* variant.

**Table 1:** List of the 24 nucleotide variants identified by WES in patient III.1 germline DNA and classified as VUS.

Chromosome	dbSNP	Zygosity	Gene	Protein	HGVS cDNA level nomenclature	Read depth (infoDP)	Allele 1 reads	Allele 2 reads	VARSOME analysis	Classification	GnomAD exomes non-cancer allele frequency	GnomAD exomes non-cancer allele frequency PopMax	lien varsome
1	rs145119239	Heterozygous wt/var	*TFAP2E*	p.R363C	NM_178548.3:c.1087C>T	94	41	53	PP3	VUS	3.01883e−03	4.94826e−03	varso.me/L4rN
1	rs41264582	Heterozygous wt/var	*EDEM3*	p.P192L	NM_001319960.1:c.575C>T	38	19	19	PP3	VUS	9.47575e−03	1.46391e−02	varso.me/L4rg
1	rs776729303	Heterozygous wt/var	*TMCC2*	p.A344V	NM_014858.3:c.1031C>T	149	92	57	PM2 + PP3	VUS	8.44509e−06	1.94791e−05	varso.me/L4tC
2	rs199779856	Heterozygous wt/var	*TMEM214*	p.G263S	NM_017727.4:c.787G>A	123	78	45	PP3	VUS	9.53110e−04	2.01455e−03	varso.me/L4t6
2	rs148295709	Heterozygous wt/var	*ABHD1*	p.Y372C	NM_032604.3:c.1115A>G	165	98	67	PP3	VUS	1.89069e−03	3.54258e−03	varso.me/L4th
3	rs759213356	Heterozygous wt/var	*BSN*	p.M3497T	NM_003458.3:c.10490T>C	144	71	73	BP4	VUS	2.54304e−05	5.87982e−05	varso.me/L4um
4	rs150771247	Heterozygous wt/var	*WFS1*	p.R228H	NM_006005.3:c.683G>A	93	40	53	PP2 + PP3	VUS	7.30517e−04	1.51794e−03	varso.me/ILKo
5	rs200904107	Heterozygous wt/var	*TMCO6*	p.Y153*	NM_001300980.1:c.459C>A	75	34	41	PP3	VUS	2.57003e−05	7.08015e−05	varso.me/L4uu
7		Heterozygous wt/var	*KLHL7*	p.G440D	NM_018846.4:c.1319G>A	67	35	32	PM2 + PP2 + PP3	VUS	NA	NA	varso.me/Knaj
7	rs75395437	Heterozygous wt/var	*PSPH*	p.G90S	NM_004577.3:c.268G>A	110	93	17	PP3 + BP1	VUS	1.98379e−04	1.20700e−03	varso.me/DDqg
7	rs144546424	Heterozygous wt/var	*CPA1*	p.G166D	NM_001868.3:c.497G>A	179	80	99	PP3	VUS	1.64439e−03	3.09990e−03	varso.me/KgMU
10	rs750208278	Heterozygous wt/var	*SEC24C*	p.R1033Q	NM_004922.3:c.3098G>A	34	20	14	PM2 + PP3	VUS	1.68808e−05	3.89287e−05	varso.me/L5Ea
11	rs143772626	Heterozygous wt/var	*RSF1*	p.V1120I	NM_016578.3:c.3358G>A	50	32	18	PP3	VUS	3.40055e−05	4.89438e−05	varso.me/L5Eg
12	rs61741949	Heterozygous wt/var	*FOXN4*	p.R316H	NM_213596.2:c.947G>A	44	19	25	PP3 + BS1	VUS	7.42860e−03	1.02144e−02	varso.me/L5Ej
14	rs146886719	Heterozygous wt/var	*RIPK3*	p.R422*	NM_006871.3:c.1264C>T	205	117	88	PP3	VUS	1.57979e−03	3.59334e−03	varso.me/L4lf
14	rs752702606	Heterozygous wt/var	*FOXA1*	p.A232T	NM_004496.3:c.694G>A	100	48	52	PP3	VUS	4.70142e−05	1.08505e−04	varso.me/L5Eq
14	rs201974904	Heterozygous wt/var	*TMEM251*	p.Met114Thr	NM_001098621.3:c.341T>C	89	49	40	PM2 + PP3	VUS	NA	NA	varso.me/L5FI
15	rs367984298	Heterozygous wt/var	*TRPM1*	p.P798S	NM_001252020.1:c.2392C>T	38	20	18	PM2 + PP3	VUS	NA	NA	varso.me/L4lv
15	rs201044858	Heterozygous wt/var	*C2CD4B*	p.P27R	NM_001007595.2:c.80C>G	70	40	30	PP3 + BS1	VUS	5.00137e−03	4.33071e−03	varso.me/L5F2
15	rs180716407	Heterozygous wt/var	*PKM*	p.S5*	NM_001316318.1:c.14C>G	92	55	37	PVS1 + BP4	VUS	7.04204e−03	1.22892e−02	varso.me/L5F5
16	rs151150316	Heterozygous wt/var	*ABCC12*	p.Q831*	NM_033226.2:c.2491C>T	44	22	22	PP3	VUS	2.41188e−03	4.65328e−03	varso.me/KvbT
19	rs772237909	Heterozygous wt/var	*PNKP*	p.N461S	NM_007254.3:c.1382A>G	67	36	31	PM2 + PP3 + BP1	VUS	0.00000e+00	NA	varso.me/L5Fp
22	rs201399007	Heterozygous wt/var	*SBF1*	p.R1308W	NM_002972.3:c.3922C>T	92	45	47	PP3	VUS	6.46441e−04	1.91916e−03	varso.me/FsDo
X	rs140505250	Heterozygous wt/var	*ARR3*	p.P351R	NM_004312.2:c.1052C>G	102	50	52	PP3	VUS	1.59134e−03	2.69648e−03	varso.me/L5Fw

**Table 2:** Distribution of the five VUS in the germline DNA of the different family members.

Gene	*TMEM214*	*KLHL7*	*PKM*	*RIPK3*	*TFAP2E*
Nucleotide variant	c.787G>A	c.1319G>A	c.14C>G	c.1264C>T	c.1087C>T
Protein change	p.G263S	p.G440D	p.S5*	p.R422*	p.R363C
Patients					
III.1	+	+	+	+	+
III.2	+	Wild type	+	+	Wild type
III.3	Wild type	+	Wild type	Wild type	Wild type
II.1	+	+	+	+	+
II.2	Wild type	Wild type	Wild type	Wild type	Wild type
II.3	Wild type	Wild type	Wild type	Wild type	Wild type
I.1	Wild type	Wild type	Wild type	Wild type	Wild type

**Figure 4:**
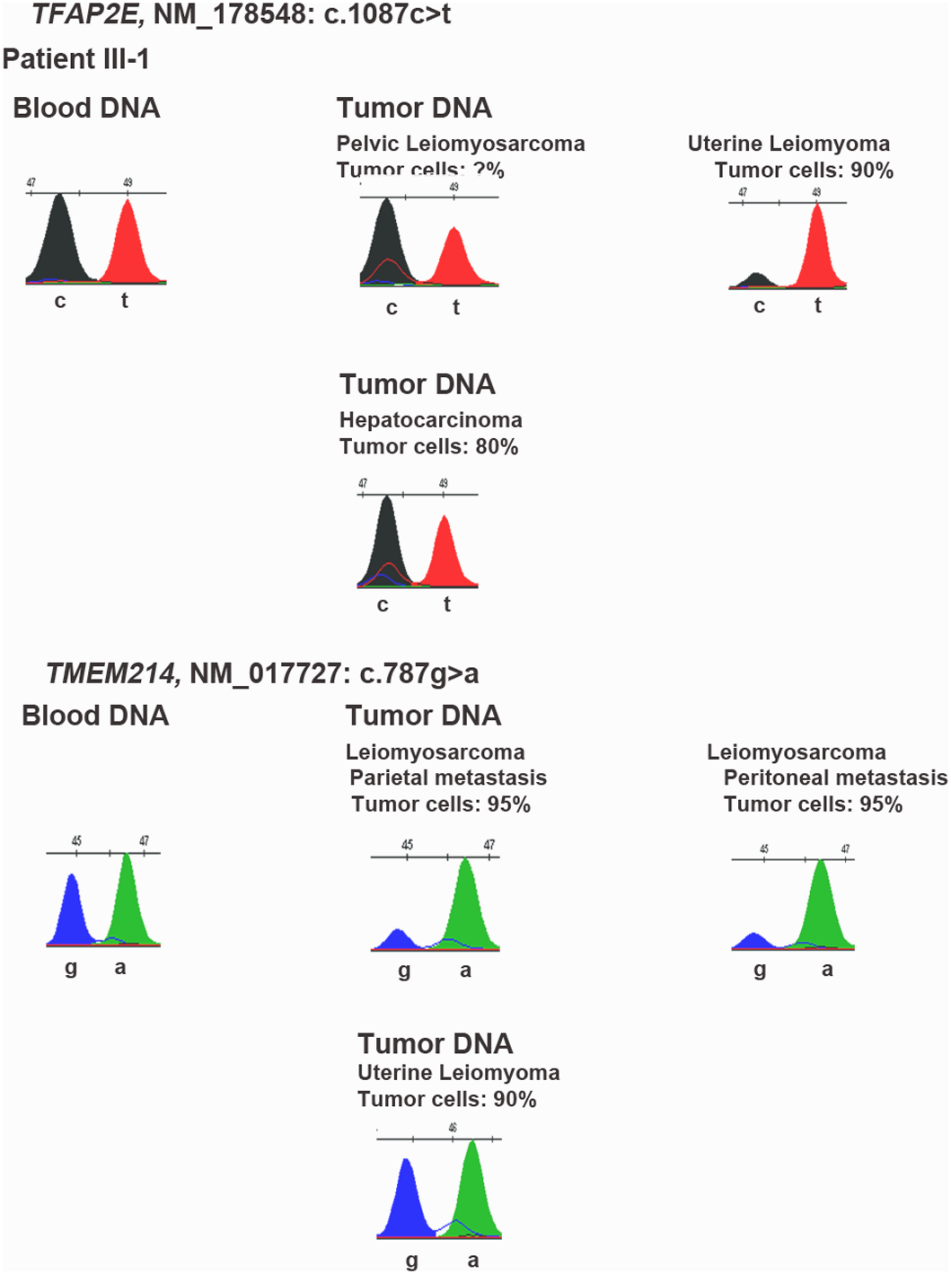
Search for a LOH of TFAP2E and TMEM214 in the DNA extracted from the different tumors from patient III.1 by SnapShot analysis.

Tumor DNA from eight different FFPE tumors from the proband (Supplementary Table 1) were analyzed by SnapShot to screen for LOH of the candidate genes. No LOH of *KLH7* WT allele was found in the PCC of the proband nor in those of her brother III.3 (Fig. 3). Unfortunately, the data were not informative for *PKM* (not shown). Regarding *TFAP2E*, LOH of the WT allele was found in the DNA from the uterine leiomyoma whereas no LOH occurred in the parietal and peritoneal metastasis of the leiomyosarcoma (Fig. 4). By contrast, a LOH of the WT allele of *TMEM214* was found in the parietal and peritoneal metastasis of the leiomyosarcoma (Fig. 4) whereas the data were not informative in the hepatocarcinoma (not shown).

## Discussion

The occurrence of a bilateral PCC at a young age in a relative (patient III.3) who did not carry the germline *KIF1B* c.4442G>A variant suggested that other susceptibility gene(s) might be implicated in the PCC/PGL predisposition of this family. Indeed, analysis of a panel of 11 classic susceptibility genes for PCC/PGL (17,18) by NGS led to the identification of a heterozygous variant of *MAX:* c.145T>C, p.Ser49Pro. This variant was also found in the germline of the two other individuals with PCC in this family, III-1 and I-1. This variant has not been previously found in the germline of patients with PCC/PGL or at the somatic level based on the LOVD Leiden and TCGA database, respectively. Using the pathogenicity criteria proposed by the ACMG, this variant was classified as a VUS rendering the interpretation more complex. However, several elements are in favor of its pathogenic role. First, the secretory pattern of the tumors of patient III-3 and I-1 is compatible with those classically observed in patients with *MAX* pathogenic variants (20). Secondly, the loss of the WT allele of* MAX* in both PCCs of III-3 suggests the role of MAX gene and causality of the p.Ser49Pro variant in the pathogenesis of the PCCs. Thirdly, the introduction of a proline at position 49 seems incompatible with a native 3D conformation for the MAX protein. In addition, although histology of the adrenal nodule identified in patient II-3 is presently not available, its imaging pattern is compatible with a PCC. If confirmed in the follow-up, this diagnosis provides support to the pathogenic role of the* MAX* variant since II-3 is WT for *KIF1B*.

The interpretation of the pathogenesis of the PCCs occurring in the proband and her grandfather is more intricate. They both have the *MAX* p.Ser49Pro plus the* KIF1B* p.Ser1481Asn germline variants. We were unable to identify LOH of *KIF1B* (1) or *MAX* WT alleles in the proband’s PCC despite the analyses of multiple separate fragments from the most recently removed PCC which was not fixed in Bouin’s reagent known to be deleterious for DNA (21). However, as discussed previously, our additional data on this tumor does not allow us to rule out the contribution of high levels of nontumoral tissue to the lack of detectable LOH. Although we cannot exclude that both *KIF1B* and *MAX* may contribute to the PCC phenotype or to the clinical variability in this family, *KIF1B* pathogenic variants have rarely been described in patients with PCC/PGL since our initial report in 2008 (1, 7, 8). Only one, a p.Tyr835Cys variant, was reported by Welander *et al.* in a 54-year-1old woman with a unilateral PCC and an endometrial carcinoma (7). This patient had no germline variants in any of the 11 other major susceptibility genes for PCC/PGL but no somatic LOH of the wild-type allele was found, so preventing any definitive conclusion on the pathogenic relevance of this novel variant.

Given the phenotypic variability of this family, with multiple non-PCC/PGL cancers in patient III.1, and to evaluate the possibility that other susceptibility events were at play, we performed WES on the germline DNAs from patient III.1 and II.2. Our working model was that patient III.1 had *de novo* or maternally-inherited mutations in new/known susceptibility genes for hereditary cancers or, alternatively, in ‘modifier genes’ which could have an impact on cancer-promoting pathways (24). These additional nucleotide variants, combined with the* KIF1B* and *MAX* germline variants, might explain the very severe phenotype of this patient. WES excluded a pathogenic mutation in other genes predisposing to familial PCC/PGL and also in 30 other hereditary cancer susceptibility genes (22). Among the 125 variants which were private to the proband most were classified as benign or probably benign and were filtered out; the 24 remaining variants were all classified as VUS. Based on bibliographic data, tissue pattern of expression, biological function of the encoded protein, and a catalog of somatic mutations, we narrowed down the list to five variants in five different genes which may be implicated either in the pathogenesis of PCC (*KLHL7* and *PKM*) or sarcomas (*RIPK3*, *TMEM214* and *TFAP2E*). We did not detect a LOH of the WT allele of *KLHL7* or *PKM* in the proband’s PCC. Thus, we cannot assign any causality in the pathogenesis of the proband’s PCCs to one of these variants.

We propose that in this family the genetic susceptibility to PCC/PGL is linked to the *MAX* nucleotide variant which is however associated with an incomplete penetrance since patient II-2 did not develop any symptomatic adrenal lesion. The KIF1B isoform β p.Ser1481Asn variant, which is partly defective in the apoptotic culling of neural crest progenitors, may also contribute to the occurrence of NB in the childhood of patient III-1, similar to other rare observations in NB (3). Finally, the leiomyosarcoma and hepatic carcinoma of the proband, and her father’s lung adenocarcinoma may be MAX and KIF1B independent. The involvement of *TFAP2E* and *TMEM214* variants is an attractive hypothesis but the pathways acting in disease development remain to be determined.

## Supplementary Material

TableS1 - List of the tissue samples from the proband III-1 analyzed by SnapShot to identify a LOH

Table S2- List of the VUS identified by NGS in 3 separate fragments from the left PCC of the proband III-1

Figure S1

Figure S2

Figure S3

## Declaration of interest

The authors declare that there is no conflict of interest that could be perceived as prejudicing the impartiality of the research reported.

## Funding

This work was supported in part by the Association de Recherche en Endocrinologie et Métabolismes (grant to P P).
