# Supplementary material for: Genetic predisposition to neural crest-derived tumors: revisiting the role of KIF1B
Source: Endocr Connect. 2020 Oct 8;9(10):1042–50. doi: 10.1530/EC-20-0460 (PMC7707833; doi:10.1530/EC-20-0460)
Supplement: Figure S1 [file supplementary_figure_1.pdf]

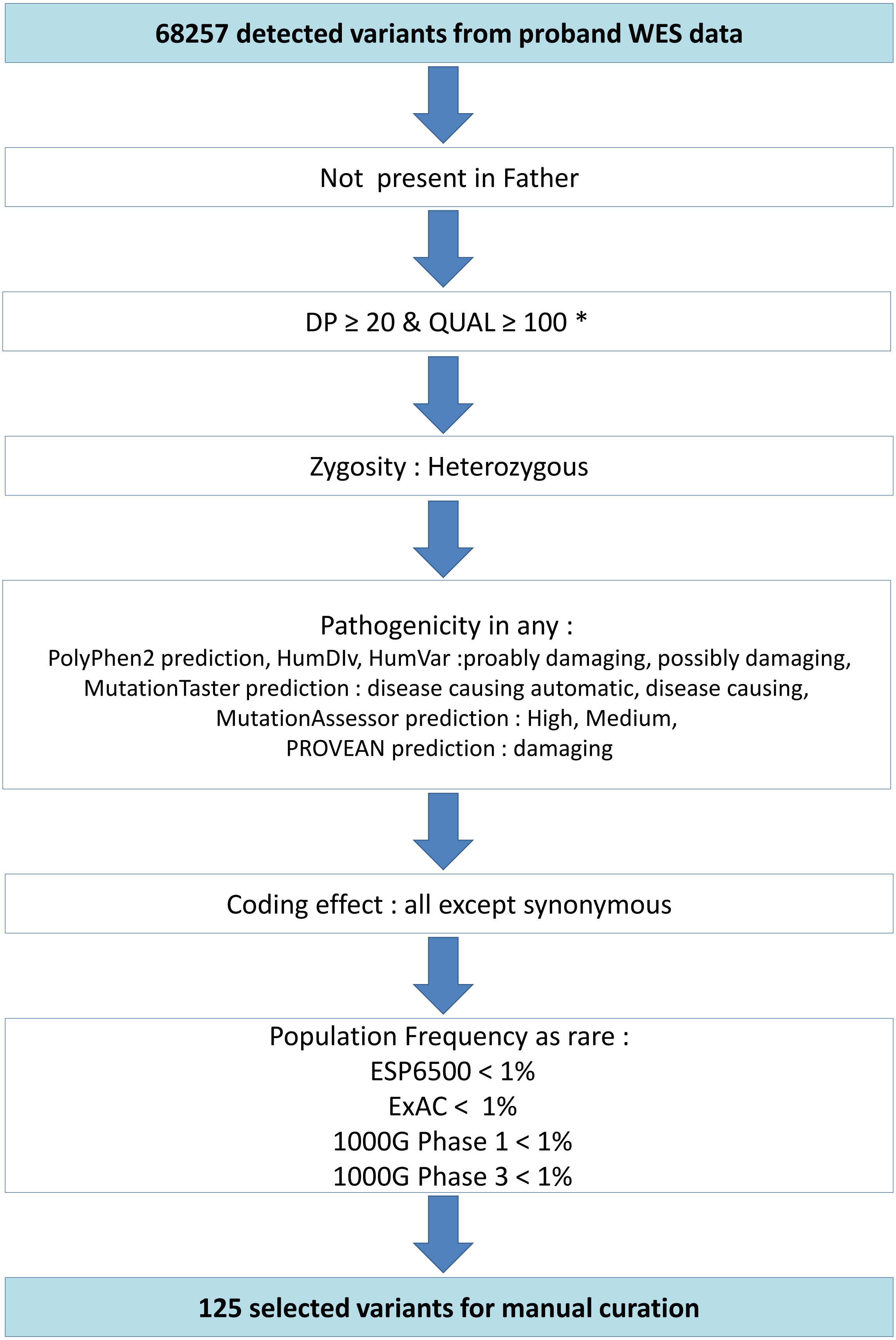

68257 detected variants from proband WES data
Not present in Father
DP ≥ 20 & QUAL ≥ 100 *
Zygosity : Heterozygous
Pathogenicity in any :
PolyPhen2 prediction, HumDIv, HumVar :proably damaging, possibly damaging,
MutationTaster prediction : disease causing automatic, disease causing,
MutationAssessor prediction : High, Medium,
PROVEAN prediction : damaging
Coding effect : all except synonymous
Population Frequency as rare :
ESP6500 < 1%
ExAC < 1%
1000G Phase 1 < 1%
1000G Phase 3 < 1%
125 selected variants for manual curation
