# Supplementary material for: Genetic predisposition to neural crest-derived tumors: revisiting the role of KIF1B
Source: Endocr Connect. 2020 Oct 8;9(10):1042–50. doi: 10.1530/EC-20-0460 (PMC7707833; doi:10.1530/EC-20-0460)
Supplement: Figure S2 [file supplementary_figure_2.pdf]

Sequence	Max genotype	Individual ID	DNA source
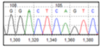	S49S (WT)	Proband's mother	germline
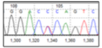	S49P	Proband's father (II-4)	germline
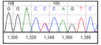	S49P	Proband's brother (III-2)	germline
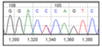	S49P	Proband (III-1)	germline
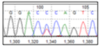	S49P	Proband's left pheo (III-1)	tumor
