# Supplementary figures and images for: Genetic predisposition to neural crest-derived tumors: revisiting the role of KIF1B
Source: Endocr Connect. 2020 Oct 8;9(10):1042–50. doi: 10.1530/EC-20-0460 (PMC7707833; doi:10.1530/EC-20-0460)

A

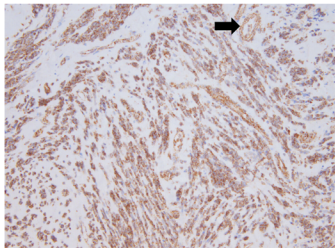

B

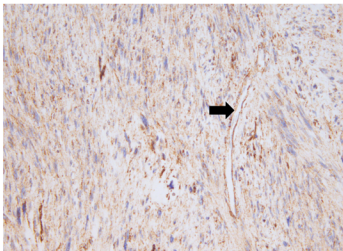

Figure S3

Supplement: Figure S3 [file supplementary_figure_3.pdf]
